# A scenario modelling analysis to anticipate the impact of COVID-19 vaccination in adolescents and children on disease outcomes in the Netherlands, summer 2021

**DOI:** 10.2807/1560-7917.ES.2022.27.44.2101090

**Published:** 2022-11-03

**Authors:** Kylie E C Ainslie, Jantien A Backer, Pieter T de Boer, Albert Jan van Hoek, Don Klinkenberg, Hester Korthals Altes, Ka Yin Leung, Hester de Melker, Fuminari Miura, Jacco Wallinga

**Affiliations:** 1 Centre for Infectious Disease Control, National Institute for Public Health and the Environment, Bilthoven, the Netherlands; 2 School of Public Health, Imperial College London, London, United Kingdom; 3 MRC Centre for Global Infectious Disease Analysis and Abdul Latif Jameel Institute for Disease and Emergency Analytics, Imperial College London, London, United Kingdom; 4 Center for Marine Environmental Studies (CMES), Ehime University, Ehime, Japan; 5 Department of Biomedical Data Sciences, Leiden University Medical Center, Leiden, the Netherlands

**Keywords:** SARS-CoV-2, COVID-19 vaccination, children and adolescents, health policy, disease outcomes, scenario modelling

## Abstract

**Background:**

Since the roll-out of COVID-19 vaccines in late 2020 and throughout 2021, European governments have relied on mathematical modelling to inform policy decisions about COVID-19 vaccination.

**Aim:**

We present a scenario-based modelling analysis in the Netherlands during summer 2021, to inform whether to extend vaccination to adolescents (12–17-year-olds) and children (5–11-year-olds).

**Methods:**

We developed a deterministic, age-structured susceptible-exposed-infectious-recovered (SEIR) model and compared modelled incidences of infections, hospital and intensive care admissions, and deaths per 100,000 people across vaccination scenarios, before the emergence of the Omicron variant.

**Results:**

Our model projections showed that, on average, upon the release of all non-pharmaceutical control measures on 1 November 2021, a large COVID-19 wave may occur in winter 2021/22, followed by a smaller, second wave in spring 2022, regardless of the vaccination scenario. The model projected reductions in infections/severe disease outcomes when vaccination was extended to adolescents and further reductions when vaccination was extended to all people over 5 years-old. When examining projected disease outcomes by age group, individuals benefitting most from extending vaccination were adolescents and children themselves. We also observed reductions in disease outcomes in older age groups, particularly of parent age (30–49 years), when children and adolescents were vaccinated, suggesting some prevention of onward transmission from younger to older age groups.

**Conclusions:**

While our scenarios could not anticipate the emergence/consequences of SARS-CoV-2 Omicron variant, we illustrate how our approach can assist decision making. This could be useful when considering to provide booster doses or intervening against future infection waves.

**Key public health message**

**What did you want to address in this study?**

Scenario modelling enables policy makers to consider a range of possible future outcomes of an event in their decisions. We wished to guide a policy decision to extend the COVID-19 vaccination programme to adolescents and children in the Netherlands in summer 2021, should new infection waves occur in late 2021 and early 2022.

**What have we learnt from this study?**

Our scenario modelling, done before the SARS-CoV-2 Omicron variant appeared, projected that extending vaccination to adolescents would reduce the rates of infections and severe disease in the population and extending it to children under 12 years old, would reduce these rates further. However, vaccination alone would not prevent future COVID-19 waves.

**What are the implications of your findings for public health?**

Vaccination for children and adolescents was recommended while other measures such as mask-wearing remained. In hindsight, our approach was important, considering that Omicron emerged in late November 2021 and subsequent infection waves occurred. Scenario modelling can support future policy decisions on COVID-19 vaccine booster doses or inform on what one might expect in the event of the emergence of other variants.

## Introduction

Since the roll-out of coronavirus disease (COVID-19) vaccines in late 2020 and throughout 2021 [1], European governments have relied on mathematical modelling to inform policy decisions about COVID-19 vaccination [2]. Examples include how to best allocate limited numbers of vaccines to achieve maximum impact, if vaccination should be extended beyond adults (≥ 18 years old), and when and who to re-vaccinate (boost) [3]. Scenario modelling, in an infectious disease context, aims to provide long-term projections of epidemic trajectories under different scenarios [4], and can provide useful insight about the likely direction and magnitude of change (between scenarios) and the trade-offs between different interventions [5]. Unlike, forecasting, which aims to predict what will happen in a short time frame (typically, a few weeks) [6,7], scenario modelling usually covers many months [8], providing bounds for outbreak trajectories. This provides policymakers with more insight and perspective to make decisions, which are usually most effective with regards to epidemics if they can be made before the modelled scenarios actually occur.

In this work, we present an analysis to inform a policy decision during summer 2021, specifically whether to extend vaccination to adolescents (12–17-year-olds) and children (5–11-year-olds). To this end, we developed a deterministic, age-structured susceptible-exposed-infectious-recovered (SEIR) model. We briefly describe the debate surrounding this policy decision to motivate this analysis and then present the results of our scenario modelling. Finally, we discuss the implications of our findings and reflect on our modelling conclusions in light of the emergence of the Omicron (Phylogenetic Assignment of Named Global Outbreak (Pango) lineage: B.1.1.529) variant. 

### Motivation and setting

In the summer months of 2021 in the northern hemisphere, following the roll-out of COVID-19 vaccination programmes and the achievement of high COVID-19 vaccination coverage among adults in high-income countries, there was concern over future waves of severe acute respiratory syndrome coronavirus 2 (SARS-CoV-2) infections. In the Netherlands, COVID-19 vaccines had been approved for adolescents [9] and children [10], but not yet administered to healthy members of these two population groups. One of the main policy decisions under consideration in the country was the extension of COVID-19 vaccination to healthy adolescents due to the risk of increased transmissibility during the winter months [11], waning natural immunity and vaccine-induced protection [12], and reduced vaccine effectiveness against new variants [13]. According to a report from the European Centre for Disease Prevention and Control (ECDC), up to 6 May 2021, 12 countries in the European Union/European Economic Area (EU/EEA) had started to vaccinate individuals under 18 years-old [14]. In an effort to limit severe disease incidence in these young people, half of the 12 countries targeted for vaccination, either individuals with underlying conditions, or in healthcare facilities, or vulnerable/at risk of severe COVID-19 outcomes [14]. In the Netherlands, as in most countries, the decision to vaccinate healthy adolescents and children took into account the potential risks/benefits for these age groups, as well as indirect positive effects on other groups in the population [3].

Some vaccinated adolescents may experience adverse events, such as myocarditis (heart inflammation), following vaccination with a COVID-19 vaccine [15,16]; however, occurrence of myocarditis in adolescents is rare. A study evaluating 192,405,448 persons receiving a total of 354,100,845 mRNA-based COVID-19 vaccines in the United States (US) from December 2020 to August 2021 found that the rate of myocarditis was 70.7 per million doses of the Comirnaty (BNT162b2, BioNTech-Pfizer, Mainz, Germany/New York, US) vaccine in adolescent males aged 12–17 years and 105.9 per million doses of the BNT162b2 vaccine in adolescent males aged 16–17 years [16]. Healthy adolescents and children can be infected by and transmit SARS-CoV-2 [17], but are much less likely to experience severe disease [18,19] and die [20] following infection with SARS-CoV-2 compared with adults. Despite their reduced risks of severe outcomes, adolescents and children may experience symptoms lasting months after infection (‘long COVID’) [21]. Estimates of the prevalence of long-COVID in children and adolescents range from 0% [22,23] to 27% [21]. Excluding long COVID, the disease burden of COVID-19 in 2021 among adolescents and children in the Netherlands has been shown to be similar to seasonal influenza [3], while in the US, hospitalisation rates in adolescents and children due to COVID-19 between March 2020 and December 2021 were similar to or higher than those from the 2017/18, 2018/19, and 2019/20 influenza seasons [24-26]. Therefore, a direct benefit of vaccinating adolescents and children is to reduce incidence of severe disease and long COVID, as well as infections, in these age groups.

Another objective for vaccinating adolescents and children is to reduce transmission from these groups to other, more vulnerable, groups. Adolescents and children make a high number of daily contacts [27]; therefore, they are likely to be larger contributors to transmission compared to older adults during outbreaks, as was seen in late June and early July 2021 in the Netherlands [28]. In the United Kingdom (UK), an increase in infections in adolescents and young adults preceded the second and third COVID-19 pandemic waves, where infections later spread to older age groups [29].

In this work, using the Netherlands as an example, we present an analysis anticipating the quantitative impacts of vaccinating adolescents and children, on SARS-CoV-2 infection and disease outcomes (e.g. hospital admissions, and intensive care (IC) admissions), before the emergence of the Omicron variant. We compare the obtained incidences of disease outcomes in extended vaccination scenarios to those when only adults are vaccinated.

## Methods

### Model description

We developed a deterministic age-structured compartmental SEIR model extended to include states for severe disease outcomes and vaccination status. The population was partitioned into 10-year age groups (0–9, 10–19, …, 70–79, ≥ 80). Within each age group we further stratified the population into those who were unvaccinated, separately, those who were vaccinated with one to five doses, and then finally into disease states: susceptible (S), infected but not yet infectious (E), infectious (I), hospitalised (H), in intensive care (IC), returned to the hospital ward after intensive care (H_IC_), recovered (R), and dead (D) (Supplementary Figure S1; Basic conceptual model diagram). The model considered that when a person is vaccinated, they first enter a hold state where they are vaccinated, but not yet (fully) protected. After a delay period, they enter the vaccinated and protected state for the dose they have received. In the model, natural immunity to infection wanes by 60% after 8 months [30] and follows an Erlang distribution. Therefore, individuals who have recovered transition back to the susceptible compartment. Only susceptible individuals can enter a vaccinated compartment.

The model is designed to incorporate a single vaccine product with up to five doses that (i) reduces susceptibility to infection, (ii) reduces risk of hospitalisation if a vaccinated individual is infected, and (iii) reduces risk of infecting others (transmission) if a vaccinated person is infected. The vaccine provides ‘leaky’ protection (i.e. the vaccine reduces the probability of infection and severe disease in vaccinated individuals). We incorporate different vaccine products by taking the daily weighted average of the number of people with each vaccine product (and dose), the corresponding delay to protection of each vaccine product, and the vaccine effectiveness against each outcome (Supplementary Table S1; Vaccine effectiveness for each vaccine by dose based on observational studies). Rate of vaccination by vaccine product and dose is a model input (Supplemental Material; Outcome equations).

The model uses different contact matrices from the Pienter Corona Study [27,31,32] estimated in April 2017 and throughout 2020 and 2021 to approximate contact patterns under different levels of non-pharmaceutical interventions within and between age groups (Supplementary Table S2; Timeline of measures and advice during the COVID-19 outbreak in the Netherlands). These contact matrices are converted to transmission matrices by multiplying rows and columns by estimates of the relative susceptibility and infectiousness of each age group compared with the 0–9-year-old age group (Supplementary Table S4; Age-dependent model parameters).

To account for the seasonal pattern of SARS-CoV-2 transmission whereby, transmission is lower in summer and higher in winter, we define the transmission rate at time *t*, *β*(t), as a sinusoidal function of seasonality [11] (Supplemental Material; Outcome equations).

### Model fit

The baseline (non-seasonal) transmission rate *β_0_* and initial conditions for forward simulations are estimated by fitting the model to daily cases from the national notification database Osiris from 01 January 2020 to 22 June 2021, when vaccination in 12–17-year-olds began in the Netherlands (Supplementary Figure S2; Fit to case notification data). The model is fitted to data piecewise to incorporate the different non-pharmaceutical interventions within each time window (Supplementary Table S2; Timeline of measures and advice during the COVID-19 outbreak in the Netherlands). We estimate the baseline transmission rate *β_0_* within each time window using maximum likelihood estimation. We assume daily cases follow a negative binomial distribution with mean µ and overdispersion parameter ϕ.

### Scenarios

We compare three different vaccination scenarios: vaccination of adults only (≥ 18 years), vaccination of adults and adolescents (≥ 12 years) and vaccination of adults, adolescents, and children (≥ 5 years). Vaccination of children and adolescents does not impact the continued vaccination of other groups. In the scenarios in which 12–17-year-olds are vaccinated, adolescents receive the Comirnaty and Spikevax (mRNA-1273, Moderna, Cambridge, United States) vaccines beginning 22 June 2021 and reach an overall coverage of 75% by 23 August 2021 as per the Dutch vaccination distribution schedule (Supplementary Figure S3; Vaccination coverage over time by dose, vaccine type, and age group for the different vaccination scenarios). When we hypothetically extend vaccination to 5–11-year-olds (≥ 5 years), vaccination of 5–11-year-olds starts on 24 October 2021 with an allocation of 50,000 doses per day reaching a final vaccination coverage of 75%. Children receive Comirnaty.

### Simulations

Forward simulations are performed from 22 June 2021 to 31 March 2022 with the initial conditions (i.e. the number of people in each compartment when the simulation begins) based on the last day of the model fitting. The simulations begin with the same baseline transmission rate that was estimated from the last fitted time window (5 June to 22 June) and with contact patterns estimated during June 2021. All non-pharmaceutical control measures are relaxed on 1 November 2021 and not reimplemented. Therefore, the contact patterns change to those estimated pre-COVID-19 pandemic in April 2017. We use a value for the baseline transmission rate in the absence of other non-pharmaceutical interventions that is consistent with the basic reproduction number of the Delta (Pango lineage: B.1.617.2) variant (R_0_ = 5.08, *β_0_* = 0.00087) [33]. To incorporate uncertainty in the transmission rate, *β_0_* is drawn from a normal distribution with mean 0.00087 and standard deviation 7.11e-6 (corresponding to the estimated standard deviation of *β_0_* from the last time window during model fit).

We perform 200 simulations for each vaccination scenario, and sample from the posterior distribution of the contact matrices to incorporate uncertainty regarding contact patterns [34]. We simulate infections, hospital admissions, IC admissions, and deaths for each vaccination scenario. We calculate the cumulative sum of each outcome per 100,000 people for the entire simulation period. We calculate the absolute and per cent differences in cumulative sum of each disease outcome for the different vaccination scenarios (≥ 12 years and ≥ 5 years) compared with vaccination in adults only (≥ 18 years). Due to the stratification of the model population in 10-year age bands, we cannot separate 12–17-year-olds from the 10–19-year age group or 5–11-year-olds from the 0–9 and 10–19-year age groups; therefore, we report the effects of the different vaccination strategies separately for each age group.

The model is coded in R 4.1.0 [35] as a system of ordinary differential equations (Supplemental Material; Model equations). Model input parameters are shown in Supplementary Table S3 (Model parameters that do not vary with age) and Supplementary Table S4 (Age-dependent model parameters). Code is available on GitHub as an R package vacamole (https://github.com/kylieainslie/vacamole).

## Results

**Figure 1:**
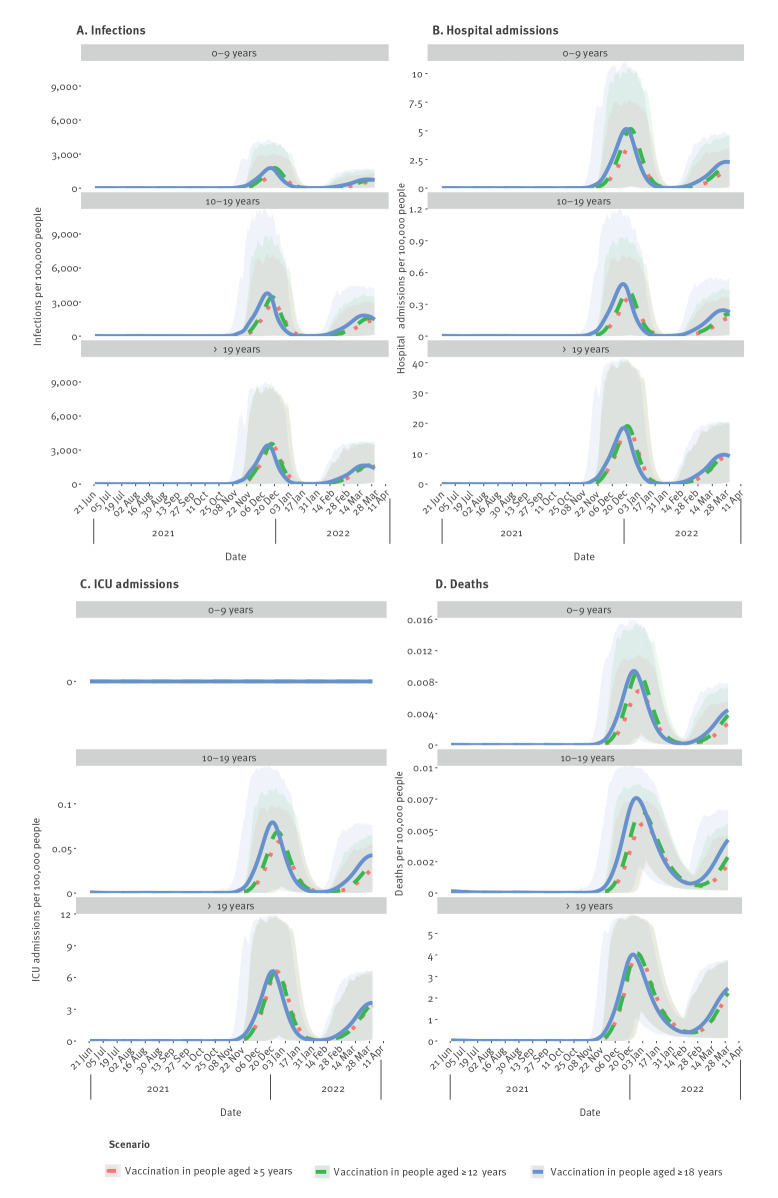
Model projected daily (A) infections, (B) hospital admissions, (C) intensive care admissions, and (D) deaths, for each vaccination scenario^a^, by age group^b^ for the Netherlands, simulation period of 22 June 2021–31 March 2022

There was considerable variability in modelled trajectories of infections in the different age groups, regardless of vaccination scenario, but on average there was a large wave of infections from late November 2021 to early January 2022 after non-pharmaceutical control measures were released on 1 November 2021, followed by a second, smaller wave in spring (March/April) 2022 (Figure 1A). The trajectories for incidences of severe disease outcomes were similar to those for infections (Figure 1B – D). We observed a delay and reduction in peak incidence in all age groups when vaccination was extended beyond adults (Table). Reductions in peak incidence were greatest in 0–9- and 10–19-year-olds. Only small reductions were observed in individuals aged greater than 19 years. A longer delay and reduction in peak incidence was seen when all individuals aged 5 years and above were vaccinated compared to when only individuals aged 12 years and above were vaccinated (Figure 1A–D).

ICU: intensive care unit.

^a^ Vaccination in people aged ≥ 5 years, ≥ 12 years, ≥ 18 years.

^b^ 0–9-year-olds, 10–19-year-olds and  > 19-year-olds.

Lines represent mean trajectory from 200 simulations and shaded regions represent the 2.5^th^ and 97.5^th^ quantiles. Contacts change from 1 November 2021 consistent with non-pharmaceutical interventions being relaxed. Y-axes for severe disease outcomes (hospital admissions, ICU admissions, and deaths) are different across age groups and care should be taken when comparing down each column (B‒D). Additionally, y-axes are different across disease outcomes, so care should be taken comparing across rows.

**Table:** Absolute and per cent difference of cumulative modelled disease outcomes per 100,000 people comparing vaccination in adults  (≥ 18 years old) only, with vaccination in ≥ 5 year-olds and vaccination in ≥ 12 year-olds, respectively, the Netherlands, cumulative disease outcomes calculated from 22 June 2021–31 March 2022

Vaccination scenario	Disease outcome	Age group	Absolute difference (95% CI)	Per cent difference (95% CI)
Vaccination in ≥ 5 year-olds	Infections	**0–9**	**−20,683.0 (−31,186.0 to −12,131.0)**	**−30.4 (−41.2 to −23.4)**
		**10–19**	**−45,308.0 (−68,890.0 to −25,118.0)**	**−30.3 (−45.0 to −18.7)**
		**20–29**	**−11,966.0 (−43,497.0 to −102.0)**	**−8.1 (−29.6 to −0.1)**
		**30–39**	**−11,084.0 (−33,800.0 to −1,282.0)**	**−8.9 (−26.7 to −1.0)**
		**40–49**	**−11,494.0 (−31,856.0 to −1,073.0)**	**−9.2 (−26.5 to −1.0)**
		**50–59**	**−10,724.0 (−31,024.0 to −842.0)**	**−8.6 (−26.0 to −1.0)**
		**60–69**	**−10,362.0 (−34,265.0 to 384.0)**	**−7.6 (−25.8 to 0.3)**
		**70–79**	**−9,596.0 (−32,182.0 to 895.0)**	**−7.1 (−24.8 to 0.6)**
		**≥ 80**	**−9,831.0 (−37,006.0 to 1,486.0)**	**−6.7 (−26.0 to 0.9)**
	Hospital admissions	**0–9**	**−64.2 (−98.0 to −37.7)**	**−32.4 (−44.0 to −24.8)**
		**10–19**	**−6.8 (−10.1 to −3.8)**	**−34.6 (−46.3 to −24.1)**
		**20–29**	**−4.6 (−14.3 to −0.2)**	**−8.9 (−25.9 to −0.3)**
		**30–39**	**−14.9 (−43.3 to −0.7)**	**−9.4 (−26.6 to −0.4)**
		**40–49**	**−29.2 (−83.1 to −2.2)**	**−9.6 (−26.9 to −0.7)**
		**50–59**	**−57.9 (−179.0 to −1.9)**	**−9.1 (−26.7 to −0.3)**
		**60–69**	**−88.9 (−297.0 to 2.4)**	**−8.2 (−25.6 to 0.2)**
		**70–79**	**−149.0 (−520.0 to 12.5)**	**−7.6 (−25.3 to 0.5)**
		**≥ 80**	**−182.0 (−616.0 to 19.4)**	**−7.4 (−24.9 to 0.8)**
	IC admissions	**0–9**	**0.0 (0.0 to 0.0)**	**NA**
		**10–19**	**−1.2 (−1.7 to −0.7)**	**−34.1 (−44.5 to −26.0)**
		**20–29**	**−1.2 (−3.2 to −0.1)**	**−8.9 (−23.8 to −0.6)**
		**30–39**	**−4.1 (−11.6 to −0.4)**	**−9.1 (−23.4 to −1.0)**
		**40–49**	**−10.9 (−30.0 to −1.2)**	**−9.4 (−23.8 to −1.1)**
		**50–59**	**−25.3 (−74.6 to −1.7)**	**−8.9 (−23.7 to −0.8)**
		**60–69**	**−44.8 (−139.0 to −0.1)**	**−8.1 (−23.0 to 0.0)**
		**70–79**	**−64.3 (−204.0 to 4.0)**	**−7.6 (−23.1 to 0.4)**
		**≥ 80**	**−18.6 (−58.0 to 0.8)**	**−7.3 (−22.3 to 0.3)**
	Deaths	**0–9**	**−0.1 (−0.2 to −0.1)**	**−31.8 (−41.7 to −25.5)**
		**10–19**	**−0.1 (−0.2 to −0.1)**	**−32.0 (−39.5 to −26.3)**
		**20–29**	**−0.1 (−0.3 to 0.0)**	**−72.7 (−18.5 to −0.6)**
		**30–39**	**−0.6 (−1.5 to −0.1)**	**−7.6 (−18.7 to −1.2)**
		**40–49**	**−1.3 (−3.4 to −0.2)**	**−7.7 (−18.8 to −1.0)**
		**50–59**	**−4.1 (−11.1 to −0.4)**	**−7.2 (−18.5 to −0.8)**
		**60–69**	**−29.8 (−83.8 to −0.5)**	**−6.1 (−16.5 to −0.1)**
		**70–79**	**−41.8 (−124.0 to 2.8)**	**−6.4 (−17.7 to 0.4)**
		**≥ 80**	**−60.0 (−181.0 to 2.3)**	**−7.0 (−21.0 to 0.3)**
Vaccination in ≥ 12 year-olds	Infections	**0–9**	**−6,212.0 (−14,504.0 to −1,676.0)**	**−9.3 (−21.8 to −2.9)**
		**10–19**	**−28,643.0 (−51,316.0 to −14,960.0)**	**−19.3 (−34.0 to −10.0)**
		**20–29**	**−9,190.0 (−39,056.0 to −919.0)**	**−6.3 (−24.5 to −0.5)**
		**30–39**	**−8,484.0 (−27,668.0 to −475.0)**	**−6.9 (−24.1 to −0.3)**
		**40–49**	**−8,809.0 (−29,475.0 to −632.0)**	**−7.1 (−23.7 to −0.4)**
		**50–59**	**−8,326.0 (−27,609.0 to −506.0)**	**−6.8 (−23.5 to −0.4)**
		**60–69**	**−8,235.0 (−29,647.0 to 133.0)**	**−6.1 (−23.3 to 0.1)**
		**70–79**	**−7,759.0 (−29,535.0 to 426.0)**	**−5.8 (−22.7 to 0.3)**
		**≥ 80**	**−7,917.0 (−32,926.0 to 406.0)**	**−5.4 (−23.9 to 0.2)**
	Hospital admissions	**0–9**	**−18.9 (−44.4 to −4.6)**	**−9.5 (−22.0 to −3.2)**
		**10–19**	**−4.39 (−6.88 to −2.3)**	**−22.5 (−34.9 to −14.0)**
		**20–29**	**−3.63 (−11.7 to −0.3)**	**−7.1 (−23.6 to −0.5)**
		**30–39**	**−11.7 (−33.0 to −1.0)**	**−7.5 (−21.0 to −0.9)**
		**40–49**	**−23.0 (−65.3 to −2.0)**	**−7.7 (−21.2 to −0.9)**
		**50–59**	**−46.4 (−137.0 to −3.0)**	**−7.3 (20.7 to −0.7)**
		**60–69**	**−73.0 (−232.0 to −3.2)**	**−6.8 (−20.7 to −0.3)**
		**70–79**	**−124.0 (−398.0 to 0.3)**	**−6.5 (−20.3 to 0)**
		**≥ 80**	**−152.0 (−498.0 to 10.5)**	**−6.2 (−20.8 to 0.4)**
	IC admissions	**0–9**	**0.0 (0.0 to 0.0)**	**NA**
		**10–19**	**−0.8 (−1.2 to −0.4)**	**−22.4 (−33.2 to −15.3)**
		**20–29**	**−1.0 (−2.9 to −0.1)**	**−7.5 (−21.2 to −0.7)**
		**30–39**	**−3.3 (−8.9 to −0.3)**	**−7.5 (−20.6 to −0.9)**
		**40–49**	**−8.9 (−25.0 to −0.8)**	**−7.7 (−20.9 to −1.0**
		**50–59**	**−20.9 (−60.8 to −1.4)**	**−7.4 (−20.7 to −0.7**
		**60–69**	**−37.9 (−121.0 to −1.3)**	**−6.9 (−20.4 to −0.3**
		**70–79**	**−55.2 (−178.0 to −0.7)**	**−6.6 (−20.4 to −0.1)**
		**≥ 80**	**−16.2 (−54.2 to −0.1)**	**−6.4 (−19.2 to 0.0)**
	Deaths	**0–9**	**0.0 (−0.1 to 0.0)**	**−8.9 (−19.0 to −3.3)**
		**10–19**	**−0.1 (−0.1 to 0.0)**	**−20.6 (−28.3 to −15.5)**
		**20–29**	**−0.1 (−0.3 to 0.0)**	**−6.2 (−15.1 to −0.7)**
		**30–39**	**−0.5 (−1.2 to 0.0)**	**−6.2 (−15.0 to −0.8)**
		**40–49**	**−1.1 (−2.9 to −0.1)**	**−6.3 (−15.2 to −1.1)**
		**50–59**	**−3.4 (−9.2 to −0.4)**	**−6.0 (−14.6 to −0.8)**
		**60–69**	**−25.8 (−73.6 to −1.6)**	**−5.3 (−13.9 to −0.4)**
		**70–79**	**−36.5 (−106.0 to −0.7)**	**−5.6 (−16.1 to −0.1)**
		**≥ 80**	**−52.5 (−170.0 to −0.5)**	**−6.2 (−18.5 to 0.0)**

CI: confidence intervals; IC: intensive care; NA: not applicable.

We report mean differences and 95% CI calculated as quantiles from 200 simulations.

**Figure 2:**
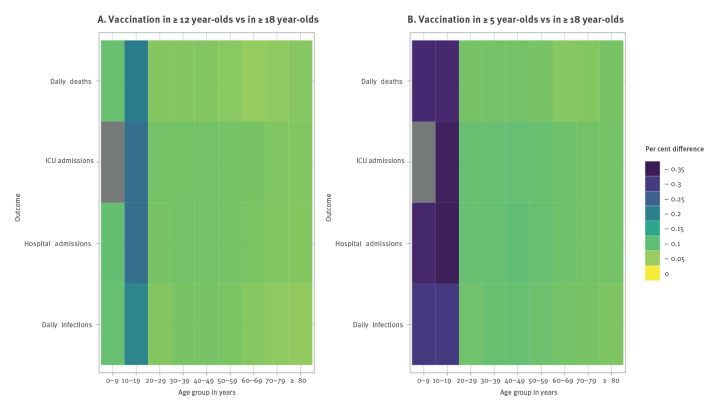
Heatmaps of mean per cent difference in cumulative disease outcomes by age group when (A) vaccination is administered in ≥ 12 year-olds compared with ≥ 18 year-olds and (B) vaccination is administered in ≥ 5 year-olds compared with adults ≥ 18 years old, the Netherlands, simulation period of 22 June 2021–31 March 2022

ICU: intensive care unit; NA: not applicable.

To determine the overall impact of extending vaccination beyond adults we calculated the cumulative number of disease outcomes per 100,000 people over the entire simulation period (22 June 2021 to 31 March 2022) and determined the per cent difference in disease outcomes comparing vaccine programme extensions (≥ 12 years and ≥ 5 years) with vaccination in only adults (≥ 18 years). We observed the greatest reduction in cumulative disease outcomes in the target groups for the vaccination programme extensions (0–9-year-olds and 10–19-year-olds) (Figure 2A-B, Table). When vaccination included individuals aged 5 years and above, we observed reductions of 30.4% (95% confidence interval (CI): 23.4% to 41.2%) in infections, 32.4% (95% CI: 24.8% to 44.0%) in hospital admissions, and 31.8% (95% CI: 25.5% to 41.7%) in deaths in 0–9-year-olds; we observed reductions of 30.3% (95% CI: 18.7% to 45.0%) in infections, 34.6% (95% CI: 24.1% to 46.3%) in hospital admissions, 34.1% (95% CI: 26.0% to 44.5%) in IC admissions, and 32.0% (95% CI: 26.3% to 39.5%) in deaths in 10–19-year-olds (Table). When vaccination included individuals aged 12 years and above, we observed more modest reductions in disease outcomes in 10–19-year-olds: 19.3% (95% CI: 10.0% to 34.0%) in infections, 22.5% (95% CI: 14.0% to 34.9%) in hospital admissions, 22.4% (95% CI: 15.3% to 33.2%) in IC admissions, and 20.6% (95% CI: 15.5% to 28.3%) in deaths (Table).

Darker colours indicate that a greater reduction in disease outcomes was observed when extending vaccination beyond adults compared with only vaccinating adults. Grey indicates an NA value.

An additional motivation for vaccinating adolescents and children is to reduce incidence of disease outcomes in the remainder of the population by preventing onward transmission. When individuals aged 5 years and above were vaccinated, we saw slightly greater reductions (ca 6–10%) in disease outcomes in the remaining age groups, particularly those of parent age (30–49 years), than when individuals 12 years and above were vaccinated (reductions of ca 5–8%) (Figure 2, Table). However, CIs of estimates of absolute differences and per cent difference in the oldest age groups contained zero, suggesting that there was little impact of vaccinating adolescents and children on disease outcomes in these groups (Table). We observed the greatest reduction in disease outcomes in hospital admissions and IC admissions in 10–19-year-olds (Figure 2, Table).

## Discussion

In this work, we performed a scenario modelling analysis to guide policy surrounding the extension of the COVID-19 vaccination programme to include adolescents and children in the Netherlands in summer 2021, before the emergence of the Omicron variant. Using the example of extending vaccination to adolescents and children we show what information can be provided by scenario modelling, while also highlighting the many uncertainties within scenario modelling. We simulated disease outcomes from 22 June 2022 to 31 March 2022 in which an event occurred in November 2021 (here, non-pharmaceutical interventions were relaxed) that may cause a new wave of infections. We compared disease outcomes between vaccination scenarios to quantify the projected impact of extending COVID-19 vaccination to adolescents and children.

Our model projections showed that, on average, upon the release of all non-pharmaceutical control measures on 1 November 2021, a large wave in COVID-19 disease outcomes may occur in winter 2021/22, followed by a smaller, second wave in spring 2022, regardless of vaccination scenario. Therefore, despite reductions in incidences of infection and various severe disease outcomes when younger age groups were included in the COVID-19 vaccination programme (Table), extension of vaccination alone would not prevent future waves of infection. These model projections indicated that future policy would have to balance vaccination with non-pharmaceutical interventions to prevent future waves of infections.

When we examined projected disease outcomes by age group we saw, unsurprisingly, that the individuals who benefitted most from extending vaccination were adolescents and children themselves (Figure 2) due to the direct protection against infection and severe disease provided by vaccination. We also observed reductions in disease outcomes in older age groups, particularly those of parent age, when children and adolescents were vaccinated, suggesting that some prevention of onward transmission from younger age groups to older age groups is a reasonable expectation if model assumptions about vaccine effectiveness against transmission are realistic. However, the prevention of onward transmission may not extend to the oldest age groups, where confidence intervals of absolute and per cent difference included zero (Table). Therefore, if the aim of policy is to protect the elderly, then vaccination of adolescents and children may not be the most effective policy decision. Previous work has highlighted that, due to the large number of contacts made by adolescents and children [27], physical distancing measures will be most effective if they are targeted at age groups that contribute most to further spread [36].

In hindsight, with the emergence of the Omicron variant in late November 2021 [37] and subsequent waves of infection in winter 2021/22 and spring 2022 [28], this model-based approach to help inform policy was extremely important. While our modelling did not endeavour to predict the emergence of a new variant or precisely when a new peak might occur, we did consider the impact of extending vaccination to adolescents and children in the event of additional waves of infections. As a result of the scenario modelling and the evolving epidemiological situation at the time, vaccination was extended to younger age groups in the Netherlands and non-pharmaceutical interventions remained in place [38].

This study has a number of limitations. The analysis presented here was performed in summer 2021 and the model assumptions reflected the available knowledge at that time, particularly based on the characteristics of the Delta variant. The model did not take into account the emergence of the Omicron variant, which was first detected in November 2021 [39]. Our projections considered the scenario whereby non-pharmaceutical interventions were relaxed and never re-implemented. In reality, if severe disease outcomes rose enough to stress healthcare systems, control measures would be re-implemented; therefore, the projections here can be viewed as an upper boundary on disease outcome projections.

In conclusion, we highlight the importance of scenario modelling to inform future policy and illustrate how scenario modelling can be used to guide policy. Looking in hindsight, we see that model projections do not predict the future but can be very helpful when considering a range of possible future outcomes. The value of scenario modelling has received growing attention and several collaborative scenario modelling hubs have recently been initiated to better inform policy in the US [4,8] and Europe [39] by harnessing projections from multiple models to better project future epidemic trajectories. These initiatives, in addition to the model and framework presented here, can continue to guide future policy decisions, such as whether and when to provide booster doses or what to expect in the event of the emergence of another variant.

## Supplementary Data

824000 Supplementary Material

## References

[1] Ritchie M, Rodés-Guirao A, Giattino O-O, et al. Coronavirus Pandemic (COVID-19). Our World in Data. Available from: https://ourworldindata.org/coronavirus

[2] Rodríguez A Kamarthi H Agarwal P Ho J Patel M Sapre S Data-Centric Epidemic Forecasting: A Survey. arXiv [cs.LG]. 2022.

[3] Ainslie K, Backer J, van Hoek AJ, Klinkenberg D, McDonald S, Miura F, et al. The expected outcome of COVID-19 vaccination strategies. Rijksinstituut voor Volksgezondheid en Milieu (RIVM); 2021 Aug. Available from: https://www.rivm.nl/documenten/expected-outcome-of-covid-19-vaccination-strategies

[4] covid19-scenario-modeling-hub: COVID-19 Scenario Modeling Hub. Github; Available from: https://github.com/midas-network/covid19-scenario-modeling-hub

[5] Greiner R Puig J Huchery C Collier N Garnett ST . Scenario modelling to support industry strategic planning and decision making. Environ Model Softw. 2014;55:120-31. 10.1016/j.envsoft.2014.01.011

[6] Cramer EY Ray EL Lopez VK Bracher J Brennen A Castro Rivadeneira AJ Evaluation of individual and ensemble probabilistic forecasts of COVID-19 mortality in the United States. Proc Natl Acad Sci USA. 2022;119(15):e2113561119. 10.1073/pnas.2113561119 35394862 PMC9169655

[7] Sherratt K Gruson H Grah R Johnson H Niehus R Prasse B Predictive performance of multi-model ensemble forecasts of COVID-19 across European nations. medRxiv. 2022; 10.7554/eLife.81916 PMC10238088 37083521

[8] Borchering RK Viboud C Howerton E Smith CP Truelove S Runge MC Modeling of Future COVID-19 Cases, Hospitalizations, and Deaths, by Vaccination Rates and Nonpharmaceutical Intervention Scenarios - United States, April-September 2021. MMWR Morb Mortal Wkly Rep. 2021;70(19):719-24. 10.15585/mmwr.mm7019e3 33988185 PMC8118153

[9] European Medicines Agency (EMA). COVID-19 vaccines: authorised. Amsterdam: EMA; 2021. [Accessed 18 Nov 2021]. Available from: https://www.ema.europa.eu/en/human-regulatory/overview/public-health-threats/coronavirus-disease-covid-19/treatments-vaccines/vaccines-covid-19/covid-19-vaccines-authorised#authorised-covid-19-vaccines-section

[10] Federal Drug Administration (FDA). Emergency Use Authorization (EUA) for an Unapproved Product Review Memorandum. October 29, 2021. Silver Spring: FDA; 2021.

[11] Liu X Huang J Li C Zhao Y Wang D Huang Z The role of seasonality in the spread of COVID-19 pandemic. Environ Res. 2021;195:110874. 10.1016/j.envres.2021.110874 33610582 PMC7892320

[12] Pouwels KB, Pritchard E, Matthews PC, Stoesser N, Eyre DW, Vihta K-D, et al. Impact of Delta on viral burden and vaccine effectiveness against new SARS-CoV-2 infections in the UK. (preprint). 2021. Available from: https://www.ndm.ox.ac.uk/files/coronavirus/covid-19-infection-survey/finalfinalcombinedve20210816.pdf 10.1038/s41591-021-01548-7 PMC8674129 34650248

[13] Lopez Bernal J Andrews N Gower C Gallagher E Simmons R Thelwall S Effectiveness of Covid-19 Vaccines against the B.1.617.2 (Delta) Variant. N Engl J Med. 2021;385(7):585-94. 10.1056/NEJMoa2108891 34289274 PMC8314739

[14] European Centre for Disease Prevention and Control (ECDC). Interim public health considerations for COVID-19 vaccination of adolescents in the EU/EEA. 1 June 2021. Stockholm: ECDC; 2021. Available from: https://www.ecdc.europa.eu/sites/default/files/documents/Interim-public-health-considerations-for-COVID-19-vaccination-of-adolescents.pdf

[15] Marshall M Ferguson ID Lewis P Jaggi P Gagliardo C Collins JS Symptomatic Acute Myocarditis in 7 Adolescents After Pfizer-BioNTech COVID-19 Vaccination. Pediatrics. 2021;148(3):e2021052478. 10.1542/peds.2021-052478 34088762

[16] Oster ME Shay DK Su JR Gee J Creech CB Broder KR Myocarditis Cases Reported After mRNA-Based COVID-19 Vaccination in the US From December 2020 to August 2021. JAMA. 2022;327(4):331-40. 10.1001/jama.2021.24110 35076665 PMC8790664

[17] European Centre for Disease Prevention and Control (ECDC). COVID-19 in children and the role of school settings in transmission - first update. Stockholm: ECDC; 2020. Available from: https://www.ecdc.europa.eu/en/publications-data/children-and-school-settings-covid-19-transmission

[18] Ludvigsson JF . Systematic review of COVID-19 in children shows milder cases and a better prognosis than adults. Acta Paediatr. 2020;109(6):1088-95. 10.1111/apa.15270 32202343 PMC7228328

[19] Preston LE Chevinsky JR Kompaniyets L Lavery AM Kimball A Boehmer TK Characteristics and Disease Severity of US Children and Adolescents Diagnosed With COVID-19. JAMA Netw Open. 2021;4(4):e215298. 10.1001/jamanetworkopen.2021.5298 33835179 PMC8035649

[20] Bhopal SS Bagaria J Olabi B Bhopal R . Children and young people remain at low risk of COVID-19 mortality. Lancet Child Adolesc Health. 2021;5(5):e12-3. 10.1016/S2352-4642(21)00066-3 33713603 PMC7946566

[21] Buonsenso D Munblit D De Rose C Sinatti D Ricchiuto A Carfi A Preliminary evidence on long COVID in children. Acta Paediatr. 2021;110(7):2208-11. 10.1111/apa.15870 33835507 PMC8251440

[22] Radtke T Ulyte A Puhan MA Kriemler S . Long-term symptoms after SARS-CoV-2 infection in children and adolescents. JAMA. 2021;326(9):869-71. 10.1001/jama.2021.11880 34264266 PMC8283661

[23] Say D Crawford N McNab S Wurzel D Steer A Tosif S . Post-acute COVID-19 outcomes in children with mild and asymptomatic disease. Lancet Child Adolesc Health. 2021;5(6):e22-3. 10.1016/S2352-4642(21)00124-3 33891880 PMC8057863

[24] Havers FP Whitaker M Self JL Chai SJ Kirley PD Alden NB Hospitalization of Adolescents Aged 12–17 Years with Laboratory-Confirmed COVID-19 — COVID-NET, 14 States, March 1, 2020–April 24, 2021. MMWR Morb Mortal Wkly Rep. 2021;70(23):851-7. 10.15585/mmwr.mm7023e1 34111061 PMC8191866

[25] Encinosa W Figueroa J Elias Y . Severity of Hospitalizations From SARS-CoV-2 vs Influenza and Respiratory Syncytial Virus Infection in Children Aged 5 to 11 Years in 11 US States. JAMA Pediatr. 2022;176(5):520-2. 10.1001/jamapediatrics.2021.6566 35188536 PMC8861895

[26] Delahoy MJ Ujamaa D Taylor CA Cummings C Anglin O Holstein R Comparison of influenza and COVID-19-associated hospitalizations among children < 18 years old in the United States-FluSurv-NET (October-April 2017-2021) and COVID-NET (October 2020-September 2021). Clin Infect Dis. 2022;ciac388. 10.1093/cid/ciac388 35594564 PMC9129156

[27] Backer JA Mollema L Vos ERA Klinkenberg D van der Klis FRM de Melker HE Impact of physical distancing measures against COVID-19 on contacts and mixing patterns: repeated cross-sectional surveys, the Netherlands, 2016-17, April 2020 and June 2020. Euro Surveill. 2021;26(8):2000994. 10.2807/1560-7917.ES.2021.26.8.2000994 33632374 PMC7908067

[28] Rijksoverheid. Coronavirus Dashboard. 7 Sep 2021. [Accessed 7 Sep 2021]. Available from: https://coronadashboard.rijksoverheid.nl/landelijk/positief-geteste-mensen

[29] Riley S Ainslie KEC Eales O Walters CE Wang H Atchison C Resurgence of SARS-CoV-2: Detection by community viral surveillance. Science. 2021;372(6545):990-5. 10.1126/science.abf0874 33893241 PMC8158959

[30] European Covid-19 Scenario Hub: Round 2. In: covid19-scenario-hub-europe. 11 Jul 2022 [Accessed 1 Aug 2022]. Available from: https://github.com/covid19-forecast-hub-europe/covid19-scenario-hub-europe

[31] Verberk JDM Vos RA Mollema L van Vliet J van Weert JWM de Melker HE Third national biobank for population-based seroprevalence studies in the Netherlands, including the Caribbean Netherlands. BMC Infect Dis. 2019;19(1):470. 10.1186/s12879-019-4019-y 31138148 PMC6537387

[32] Rijksinstituut voor Volksgezondheid en Milieu (RIVM). PIENTER Corona Study. Dutch. Bilthoven, The Netherlands: RIVM; 6 Oct 2021. [Accessed 21 Oct 2021]. Available from: https://www.rivm.nl/pienter-corona-studie

[33] Liu Y Rocklöv J . The reproductive number of the Delta variant of SARS-CoV-2 is far higher compared to the ancestral SARS-CoV-2 virus. J Travel Med. 2021;28(7):taab124. 10.1093/jtm/taab124 34369565 PMC8436367

[34] van de Kassteele J van Eijkeren J Wallinga J . Efficient estimation of age-specific social contact rates between men and women. Ann Appl Stat. 2017;11(1):320-39. 10.1214/16-AOAS1006

[35] R Core Team. R: A language and environment for statistical computing. Vienna, Austria; 2019. Available from: https://www.r-project.org/

[36] Wallinga J van Boven M Lipsitch M . Optimizing infectious disease interventions during an emerging epidemic. Proc Natl Acad Sci USA. 2010;107(2):923-8. 10.1073/pnas.0908491107 20080777 PMC2818907

[37] Viana R Moyo S Amoako DG Tegally H Scheepers C Althaus CL Rapid epidemic expansion of the SARS-CoV-2 Omicron variant in southern Africa. Nature. 2022;603(7902):679-86. 10.1038/s41586-022-04411-y 35042229 PMC8942855

[38] Coronavirus timeline. 17 Jun 2022. Dutch. [Accessed 2 Aug 2022]. Available from: https://www.rijksoverheid.nl/onderwerpen/coronavirus-tijdlijn

[39] European Centre for Disease Control and Prevention (ECDC). European Covid-19 Scenario Hub. [Accessed 27 Jun 2022]. Stockholm: ECDC. Available from: https://covid19scenariohub.eu/index.html

