# Supplementary material for: A scenario modelling analysis to anticipate the impact of COVID-19 vaccination in adolescents and children on disease outcomes in the Netherlands, summer 2021
Source: Euro Surveill. 2022 Nov 3;27(44):2101090. doi: 10.2807/1560-7917.ES.2022.27.44.2101090 (PMC9635025; doi:10.2807/1560-7917.ES.2022.27.44.2101090)
Supplement: Supplementary Material [file 2101090_SupplementaryMaterial.pdf]

Supplemental Material

A scenario modelling analysis to anticipate the impact of COVID-19 vaccination in adolescents and children on disease outcomes in the Netherlands, summer 2021

This supplementary material is hosted by Eurosurveillance as supporting information alongside the article "A scenario modelling analysis to anticipate the impact of COVID-19 vaccination in adolescents and children on disease outcomes in the Netherlands, summer 2021" on behalf of the authors who remain responsible for the accuracy and appropriateness of the content. The same standards for ethics, copyright, attributions and permissions as for the article apply. Supplements are not edited by Eurosurveillance and the journal is not responsible for the maintenance of any links or email addresses provided therein.

Methods

We developed a deterministic age-structured compartmental susceptible-exposed-infectious-recovered (SEIR) model extended to include states for severe disease outcomes and vaccination status. The population is partitioned into 10-year age groups (0-9, 10-19, …, 70-79, 80+). Within each age group we further partition the population into those who are unvaccinated, and separately for those who are vaccinated with 1 to 5 doses, and then finally into disease states: susceptible (S), infected but not yet infectious (E), infectious (I), hospitalized (H), in intensive care (IC), return to the hospital ward after intensive care (HIC), recovered (R), and dead (D) (Figure S1).

When a person is vaccinated, they first enter a hold state where they are vaccinated, but not yet (fully) protected. After a delay period, they enter the vaccinated and protected state for the dose they have received. We assume only susceptible individuals are vaccinated. We use the model to determine the number of daily cases, hospital admissions, and IC admissions under different vaccination scenarios. The mathematical equations for determining each outcome and the differential model equations are shown below. In the model equations, bold capital letters refer to matrices, bold lower case letters indicate vectors, and plain text lower case letters indicate scalars. Since model compartments are denoted with capital letters, but are vectors due to the age-stratification of the model, we denote them with a half arrow above the compartment symbol, e.g., $\vec{S}$. Parameter definitions and values are shown in Table S3 and Table S4.

Model Equations

$$\boldsymbol{\lambda} = \beta C\ N\hat{}(-1)\ (\vec{I} + \vec{I}_{v1d} + \vec{I}_{v2d})$$

$$\frac{d\vec{S}}{dt} = -\boldsymbol{\lambda}\vec{S} - \boldsymbol{\alpha_1}\vec{S} + 4\omega R^3$$

$$\frac{d\vec{S}_{hold_{1d}}}{dt} = \boldsymbol{\alpha_1}\vec{S} - \frac{1}{\delta_1}\vec{S}_{hold_{1d}} - \lambda\vec{S}_{hold_{1d}}$$

$$\frac{d\vec{S}_{v_{1d}}}{dt} = \frac{1}{\delta_1}\vec{S}_{hold_{1d}} - \eta_1\boldsymbol{\lambda}\vec{S}_{v_{1d}} - \boldsymbol{\alpha_2}\vec{S}_{v_{1d}} + 4\omega R^3_{v_{1d}}$$

$$\frac{d\vec{S}_{hold_{2d}}}{dt} = \boldsymbol{\alpha_2}\vec{S}_{v_{1d}} - \frac{1}{\delta_2}\vec{S}_{hold_{2d}} - \eta_1\boldsymbol{\lambda}\vec{S}_{hold_{2d}}$$

$$\frac{d\vec{S}_{v_{2d}}}{dt} = \frac{1}{\delta_2}\vec{S}_{hold_{2d}} - \eta_2\boldsymbol{\lambda}\vec{\boldsymbol{S}}_{v_{2d}} - \boldsymbol{\alpha_3}\vec{S}_{v_{2d}} + 4\omega R^3_{v_{2d}}$$

$$\frac{d\vec{S}_{hold_{3d}}}{dt} = \boldsymbol{\alpha_3}\vec{S}_{v_{2d}} - \frac{1}{\delta_3}\vec{S}_{hold_{3d}} - \eta_2\boldsymbol{\lambda}\vec{S}_{hold_{3d}}$$

$$\frac{d\vec{S}_{v_{3d}}}{dt} = \frac{1}{\delta_3}\vec{S}_{hold_{3d}} - \eta_3\boldsymbol{\lambda}\vec{\boldsymbol{S}}_{v_{3d}} - \boldsymbol{\alpha_4}\vec{S}_{v_{3d}} + 4\omega R^3_{v_{3d}}$$

$$\frac{d\vec{S}_{hold_{4d}}}{dt} = \boldsymbol{\alpha_4}\vec{S}_{v_{3d}} - \frac{1}{\delta_4}\vec{S}_{hold_{4d}} - \eta_3\boldsymbol{\lambda}\vec{S}_{hold_{4d}}$$

$$\frac{d\vec{S}_{v_{4d}}}{dt} = \frac{1}{\delta_4}\vec{S}_{hold_{4d}} - \eta_4\boldsymbol{\lambda}\vec{\boldsymbol{S}}_{v_{4d}} - \boldsymbol{\alpha_5}\vec{S}_{v_{4d}} + 4\omega R^3_{v_{4d}}$$
$$\frac{d\vec{S}_{hold_{5d}}}{dt} = \boldsymbol{\alpha_5}\vec{S}_{v_{4d}} - \frac{1}{\delta_5}\vec{S}_{hold_{5d}} - \eta_4\boldsymbol{\lambda}\vec{S}_{hold_{5d}}$$
$$\frac{d\vec{S}_{v_{5d}}}{dt} = \frac{1}{\delta_5}\vec{S}_{hold_{5d}} - \eta_5\boldsymbol{\lambda}\vec{\boldsymbol{S}}_{v_{5d}} + 4\omega R^3_{v_{5d}}$$

$$\frac{d\vec{E}}{dt} = \boldsymbol{\lambda}\left(\vec{S} + \vec{S}_{hold_{1d}}\right) - \sigma\vec{E} + \epsilon$$
$$\frac{d\vec{E}_{v_{1d}}}{dt} = \eta_1\boldsymbol{\lambda}\left(\vec{S}_{v_{1d}} + \vec{S}_{hold_{2d}}\right) - \sigma\vec{E}_{v_{1d}}$$
$$\frac{d\vec{E}_{v_{2d}}}{dt} = \eta_2\boldsymbol{\lambda}\left(\vec{S}_{v_{2d}} + \vec{S}_{hold_{3d}}\right) - \sigma\vec{E}_{v_{2d}}$$
$$\frac{d\vec{E}_{v_{3d}}}{dt} = \eta_3\boldsymbol{\lambda}\left(\vec{S}_{v_{3d}} + \vec{S}_{hold_{4d}}\right) - \sigma\vec{E}_{v_{3d}}$$
$$\frac{d\vec{E}_{v_{4d}}}{dt} = \eta_4\boldsymbol{\lambda}\left(\vec{S}_{v_{4d}} + \vec{S}_{hold_{5d}}\right) - \sigma\vec{E}_{v_{4d}}$$
$$\frac{d\vec{E}_{v_{5d}}}{dt} = \eta_5\boldsymbol{\lambda}\vec{S}_{v_{5d}} - \sigma\vec{E}_{v_{5d}}$$

$$\frac{d\vec{I}}{dt} = \sigma\vec{E} - (\gamma + \boldsymbol{h})\vec{I}$$
$$\frac{d\vec{I}_{v_{1d}}}{dt} = \sigma\vec{E}_{v_{1d}} - \left(\gamma + \eta_{hosp_1}\boldsymbol{h}\right)\vec{I}_{v_{1d}}$$
$$\frac{d\vec{I}_{v_{2d}}}{dt} = \sigma\vec{E}_{v_{2d}} - \left(\gamma + \eta_{hosp_2}\boldsymbol{h}\right)\vec{I}_{v_{2d}}$$
$$\frac{d\vec{I}_{v_{3d}}}{dt} = \sigma\vec{E}_{v_{3d}} - \left(\gamma + \eta_{hosp_3}\boldsymbol{h}\right)\vec{I}_{v_{3d}}$$
$$\frac{d\vec{I}_{v_{4d}}}{dt} = \sigma\vec{E}_{v_{4d}} - \left(\gamma + \eta_{hosp_4}\boldsymbol{h}\right)\vec{I}_{v_{4d}}$$
$$\frac{d\vec{I}_{v_{5d}}}{dt} = \sigma\vec{E}_{v_{5d}} - \left(\gamma + \eta_{hosp_5}\boldsymbol{h}\right)\vec{I}_{v_{5d}}$$

$$\frac{d\vec{H}}{dt} = \boldsymbol{h}\vec{I} - (\boldsymbol{i_1} + \boldsymbol{d} + \boldsymbol{r})\vec{H}$$
$$\frac{d\vec{H}_{v_{1d}}}{dt} = \eta_{hosp_1}\boldsymbol{h}\vec{I}_{v_{1d}} - (\boldsymbol{i_1} + \boldsymbol{d} + \boldsymbol{r})\vec{H}_{v_{1d}}$$
$$\frac{d\vec{H}_{v_{2d}}}{dt} = \eta_{hosp_2}\boldsymbol{h}I_{v_{2d}} - (i_1 + d + r)H_{v_{2d}}$$
$$\frac{d\vec{H}_{v_{3d}}}{dt} = \eta_{hosp_3}\boldsymbol{h}I_{v_{3d}} - (i_1 + d + r)H_{v_{3d}}$$
$$\frac{d\vec{H}_{v_{4d}}}{dt} = \eta_{hosp_4}\boldsymbol{h}I_{v_{4d}} - (i_1 + d + r)H_{v_{4d}}$$
$$\frac{d\vec{H}_{v_{5d}}}{dt} = \eta_{hosp_5}\boldsymbol{h}I_{v_{5d}} - (i_1 + d + r)H_{v_{5d}}$$

$$\frac{d\overrightarrow{IC}}{dt} = \boldsymbol{i_1}\vec{H} - (\boldsymbol{i_2} + \boldsymbol{d_{IC}})\overrightarrow{IC}$$
$$\frac{d\overrightarrow{IC}_{v_{1d}}}{dt} = \boldsymbol{i_1}\vec{H}_{v_{1d}} - (\boldsymbol{i_2} + \boldsymbol{d_{IC}})\overrightarrow{IC}_{v_{1d}}$$
$$\frac{d\overrightarrow{IC}_{v_{2d}}}{dt} = \boldsymbol{i_1}\vec{H}_{v_{2d}} - (\boldsymbol{i_2} + \boldsymbol{d_{IC}})\overrightarrow{IC}_{v_{2d}}$$
$$\frac{d\overrightarrow{IC}_{v_{3d}}}{dt} = \boldsymbol{i_1}\vec{H}_{v_{3d}} - (\boldsymbol{i_2} + \boldsymbol{d_{IC}})\overrightarrow{IC}_{v_{3d}}$$
$$\frac{d\overrightarrow{IC}_{v_{4d}}}{dt} = \boldsymbol{i_1}\vec{H}_{v_{4d}} - (\boldsymbol{i_2} + \boldsymbol{d_{IC}})\overrightarrow{IC}_{v_{4d}}$$
$$\frac{d\overrightarrow{IC}_{v_{5d}}}{dt} = \boldsymbol{i_1}\vec{H}_{v_{5d}} - (\boldsymbol{i_2} + \boldsymbol{d_{IC}})\overrightarrow{IC}_{v_{5d}}$$

$$\frac{d\vec{H}_{IC}}{dt} = \boldsymbol{i_2}\overrightarrow{IC} - (\boldsymbol{d_{H_{IC}}} + \boldsymbol{r_{ic}})\vec{H}_{IC}$$
$$\frac{d\vec{H}_{IC_{v_{1d}}}}{dt} = \boldsymbol{i_2}\overrightarrow{IC}_{v_{1d}} - (\boldsymbol{d_{H_{IC}}} + \boldsymbol{r_{ic}})\vec{H}_{IC_{v_{1d}}}$$
$$\frac{d\vec{H}_{IC_{v_{2d}}}}{dt} = \boldsymbol{i_2}\overrightarrow{IC}_{v_{2d}} - (\boldsymbol{d_{H_{IC}}} + \boldsymbol{r_{ic}})\vec{H}_{IC_{v_{2d}}}$$
$$\frac{d\vec{H}_{IC_{v_{3d}}}}{dt} = \boldsymbol{i_2}\overrightarrow{IC}_{v_{3d}} - (\boldsymbol{d_{H_{IC}}} + \boldsymbol{r_{ic}})\vec{H}_{IC_{v_{3d}}}$$
$$\frac{d\vec{H}_{IC_{v_{4d}}}}{dt} = \boldsymbol{i_2}\overrightarrow{IC}_{v_{4d}} - (\boldsymbol{d_{H_{IC}}} + \boldsymbol{r_{ic}})\vec{H}_{IC_{v_{4d}}}$$
$$\frac{d\vec{H}_{IC_{v_{5d}}}}{dt} = \boldsymbol{i_2}\overrightarrow{IC}_{v_{5d}} - (\boldsymbol{d_{H_{IC}}} + \boldsymbol{r_{ic}})\vec{H}_{IC_{v_{5d}}}$$

$$\frac{d\vec{D}}{dt} = \boldsymbol{d}(\vec{H} + \vec{H}_{v_{1d}} + \vec{H}_{v_{2d}} + \vec{H}_{v_{3d}} + \vec{H}_{v_{4d}} + \vec{H}_{v_{5d}}) + \boldsymbol{d_{IC}}(\overrightarrow{IC} + \overrightarrow{IC}_{v_{1d}} + \overrightarrow{IC}_{v_{2d}} + \overrightarrow{IC}_{v_{3d}} + \overrightarrow{IC}_{v_{4d}} + \overrightarrow{IC}_{v_{5d}}) + \boldsymbol{d_{H_{IC}}}\left(\vec{H}_{IC} + \vec{H}_{IC_{v_{1d}}} + \vec{H}_{IC_{v_{2d}}} + \vec{H}_{IC_{v_{3d}}} + \vec{H}_{IC_{v_{4d}}} + \vec{H}_{IC_{v_{5d}}}\right)$$

$$\frac{d\vec{R}}{dt} = \gamma\vec{I} + \boldsymbol{r}\vec{H} + \boldsymbol{r_{ic}}\vec{H}_{IC} - 4\omega R$$
$$\frac{d\vec{R}_{v_{1d}}}{dt} = \gamma\vec{I}_{v_{1d}} + \boldsymbol{r}\vec{H}_{v_{1d}} + \boldsymbol{r_{ic}}\vec{H}_{IC_{v_{1d}}} - 4\omega\vec{R}_{1d}$$
$$\frac{d\vec{R}_{v_{2d}}}{dt} = \gamma\vec{I}_{v_{2d}} + \boldsymbol{r}\vec{H}_{v_{2d}} + \boldsymbol{r_{ic}}\vec{H}_{IC_{v_{2d}}} - 4\omega\vec{R}_{2d}$$
$$\frac{d\vec{R}_{v_{3d}}}{dt} = \gamma\vec{I}_{v_{3d}} + \boldsymbol{r}\vec{H}_{v_{3d}} + \boldsymbol{r_{ic}}\vec{H}_{IC_{v_{3d}}} - 4\omega\vec{R}_{3d}$$
$$\frac{d\vec{R}_{v_{4d}}}{dt} = \gamma\vec{I}_{v_{4d}} + \boldsymbol{r}\vec{H}_{v_{4d}} + \boldsymbol{r_{ic}}\vec{H}_{IC_{v_{4d}}} - 4\omega\vec{R}_{4d}$$
$$\frac{d\vec{R}_{v_{5d}}}{dt} = \gamma\vec{I}_{v_{5d}} + \boldsymbol{r}\vec{H}_{v_{5d}} + \boldsymbol{r_{ic}}\vec{H}_{IC_{v_{5d}}} - 4\omega\vec{R}_{5d}$$

$$\frac{d\vec{R}^1}{dt} = 4\omega\vec{R} - 4\omega\vec{R}^1$$
$$\frac{d\vec{R}^1_{v_{1d}}}{dt} = 4\omega\vec{R}_{v_{1d}} - 4\omega R^1_{v_{1d}}$$
$$\frac{d\vec{R}^1_{v_{2d}}}{dt} = 4\omega\vec{R}_{v_{2d}} - 4\omega R^1_{v_{2d}}$$
$$\frac{d\vec{R}^1_{v_{3d}}}{dt} = 4\omega\vec{R}_{v_{3d}} - 4\omega R^1_{v_{3d}}$$
$$\frac{d\vec{R}^1_{v_{4d}}}{dt} = 4\omega\vec{R}_{v_{4d}} - 4\omega R^1_{v_{4d}}$$
$$\frac{d\vec{R}^1_{v_{5d}}}{dt} = 4\omega\vec{R}_{v_{5d}} - 4\omega R^1_{v_{5d}}$$

$$\frac{d\vec{R}^2}{dt} = 4\omega\vec{R}^1 - 4\omega\vec{R}^2$$
$$\frac{d\vec{R}^2_{v_{1d}}}{dt} = 4\omega R^1_{v_{1d}} - 4\omega R^2_{v_{1d}}$$
$$\frac{d\vec{R}^2_{v_{2d}}}{dt} = 4\omega R^1_{v_{2d}} - 4\omega R^2_{v_{2d}}$$
$$\frac{d\vec{R}^2_{v_{3d}}}{dt} = 4\omega R^1_{v_{3d}} - 4\omega R^2_{v_{3d}}$$
$$\frac{d\vec{R}^2_{v_{4d}}}{dt} = 4\omega R^1_{v_{4d}} - 4\omega R^2_{v_{4d}}$$

$$\frac{d\overrightarrow{R^2_{v_{5d}}}}{dt} = 4\omega R^1_{v_{5d}} - 4\omega R^2_{v_{5d}}$$

$$\frac{d\vec{R}^3}{dt} = 4\omega\vec{R}^2 - 4\omega\vec{R}^3$$

$$\frac{d\overrightarrow{R^3_{v_{1d}}}}{dt} = 4\omega R^2_{v_{1d}} - 4\omega R^3_{v_{1d}}$$

$$\frac{d\overrightarrow{R^3_{v_{2d}}}}{dt} = 4\omega R^2_{v_{2d}} - 4\omega R^3_{v_{2d}}$$

$$\frac{d\overrightarrow{R^3_{v_{3d}}}}{dt} = 4\omega R^2_{v_{3d}} - 4\omega R^3_{v_{3d}}$$

$$\frac{d\overrightarrow{R^3_{v_{4d}}}}{dt} = 4\omega R^2_{v_{4d}} - 4\omega R^3_{v_{4d}}$$

$$\frac{d\overrightarrow{R^3_{v_{5d}}}}{dt} = 4\omega R^2_{v_{5d}} - 4\omega R^3_{v_{5d}}$$

Outcome Equations

Daily infections = $\lambda[(\vec{S} + \vec{S}_{hold_{1d}}) + \eta_1(\vec{S}_{v_{1d}} + \vec{S}_{hold_{2d}}) + \eta_2(\vec{S}_{v_{2d}} + \vec{S}_{hold_{3d}}) + \eta_3(\vec{S}_{v_{3d}} + \vec{S}_{hold_{4d}}) + \eta_4(\vec{S}_{v_{4d}} + \vec{S}_{hold_{5d}}) + \eta_5\vec{S}_{v_{5d}}]$

Daily cases = $\sigma(\vec{E} + \vec{E}_{v_{1d}} + \vec{E}_{v_{2d}} + \vec{E}_{v_{3d}} + \vec{E}_{v_{4d}} + \vec{E}_{v_{5d}})p_{ascertainment}$

Hospital admissions = $\boldsymbol{h}(\vec{I} + \eta_{hosp_1}\vec{I}_{v_{1d}} + \eta_{hosp_2}\vec{I}_{v_{2d}} + \eta_{hosp_3}\vec{I}_{v_{3d}} + \eta_{hosp_4}\vec{I}_{v_{4d}} + \eta_{hosp_5}\vec{I}_{v_{5d}})$

IC admissions = $\boldsymbol{i_1}(\vec{H} + \vec{H}_{v_{1d}} + \vec{H}_{v_{2d}} + \vec{H}_{v_{3d}} + \vec{H}_{v_{4d}} + \vec{H}_{v_{5d}})$

Daily Deaths = $\boldsymbol{d}(\vec{H} + \vec{H}_{v_{1d}} + \vec{H}_{v_{2d}} + \vec{H}_{v_{3d}} + \vec{H}_{v_{4d}} + \vec{H}_{v_{5d}}) + \boldsymbol{d_{IC}}(\overrightarrow{IC} + \overrightarrow{IC}_{v_{1d}} + \overrightarrow{IC}_{v_{2d}} + \overrightarrow{IC}_{v_{3d}} + \overrightarrow{IC}_{v_{4d}} + \overrightarrow{IC}_{v_{5d}}) + \boldsymbol{d_{H_{IC}}}(\overrightarrow{H_{IC}} + \vec{H}_{IC_{v_{1d}}} + \overrightarrow{H_{IC_{v_{2d}}}} + \vec{H}_{IC_{v_{3d}}} + \vec{H}_{IC_{v_{4d}}} + \overrightarrow{H_{IC_{v_{5d}}}})$

The model is designed to incorporate a single vaccine product with a 2-dose regimen that 1) reduces susceptibility to infection, 2) reduces risk of hospitalization if a vaccinated individual is infected, and 3) reduces risk of infecting others (transmission) if a vaccinated person is infected. However, since there are more than one vaccine products currently licensed for use against COVID-19 in The Netherlands (the vaccines made by Pfizer/BioNTech, Moderna, AstraZeneca, and Janssen), we incorporate different vaccine products by taking the daily weighted average of the number of people with each vaccine product (and dose), the corresponding delay to protection of each vaccine product, and the vaccine effectiveness against each outcome (Table S1). Janssen is incorporated by using zero for the number of second doses at all time points. The weighted average of the VE can be expressed as:

$$VE_{wgt_{doi}}(t) = \sum_{i=1}^{4} \frac{n_{di}(t)}{\sum_{i=1}^{4} n_{di}(t)} VE_{doi},$$

where $n_{di}(t)$ is the number of vaccines given as dose *d* (d = 1, 2) from vaccine product *i* (i = 1, 2, 3, 4) at time t and VE_{doi} is the VE against outcome *o* (*o* = infection, hospitalization, transmission)*,* for dose *d*, from vaccine product *i*.

When VE is assumed to wane, we include the amount of waning as a logistic function parameterized so that after 6 months vaccine-induced protection is reduced by 50% and reduced by 100% after 1 year. The amount of waning is calculated at a given time since vaccination as

$$\omega(t) = \frac{1}{1 + e^{(-k(t-t_0))}},$$

where k is the logistic growth rate (here, k = 0.03), t is the time since vaccination in days, and t_0 is the time point (in days) where 50% reduction occurs (here, t_0=180). Then at each timepoint, VE with waning ($VE_\omega(t)$) is calculated as

$$VE_\omega(t) = VE(t) - (VE(t)\omega(t)).$$

If $VE(t)\omega(t) > VE(t)$, then $VE_\omega(t) = 0$.

Rate of vaccination for each day, vaccine product, and dose for each age group is a model input. Increase of vaccination coverage over time was based on weekly data of allocated vaccines (up to 16 June 2021) or projected available vaccines (after 16 June 2021) by vaccine type, dose number, and target group (split up between healthcare workers, residents in institutions, chronically ill, and, if not part of one of the aforementioned groups, by age in 10-years age-bands) [1]. Projected final vaccination coverages were assumed at 90% for ≥70-year-olds and residents in institutions, 85% for 60- to 69-years-olds, health care workers and high-risk individuals, and 75% for others below 60 years of age. It was assumed that available vaccines were administered immediately until a target group reached the final coverage.

To account for the seasonal pattern of SARS-CoV-2 whereby, transmission is lower in summer and higher in winter, we define the transmission rate at time t, $\beta(t)$, as a sinusoidal function of seasonality [2]:

$$\beta(t) = \beta_0 \left[1 + \beta_1 \cos\left(2\pi \frac{t}{365.25}\right)\right].$$

β_0 is a baseline (non-seasonal) transmission rate, β_1 is the amplitude of seasonal forcing, and t is the day of the year. We assume $\beta_1 = 0.14$. The baseline transmission rate β_0 and initial conditions for forward simulations are estimated by fitting the model to daily cases from the national notification database Osiris from 01 January 2020 to 22 June 2021, when vaccination in 12-17 year olds began in The Netherlands (Figure S2). The model is fitted to data piecewise to correspond with the correct contact patterns associated with different non-pharmaceutical interventions within each time window (Table S2). We directly estimate effective reproduction number (R_t) within each time window using maximum likelihood estimation. We assume daily cases follow a negative binomial distribution with mean μ and overdispersion parameter φ. Estimates of R_t are converted to transmission rate by: $\beta_0 = \frac{R_t}{\rho} * \gamma$, where γ is the inverse of the infectious period and ρ is the dominant eigen value of the product of the diagonal matrix of the number of susceptibles and the transmission matrix. The transmission matrix is determined by multiplying the rows and columns of the contact matrix by estimates of the relative susceptibility and infectiousness of each age group compared to the 0-9 years age group (Table S4).

The amplitude of seasonal forcing β_1 is estimated by linear regression:

$$\log\left(\frac{R_t}{s(t)}\right) = b_0 + b_1 X_{AH} + b_2 X_{temp} + b_3 X_{mobility} + b_4 X_{variant} + b_5 X_{day} + b_6 X_{intervention}$$

The response variable is the logarithm of the effective reproduction number R_t [3] divided by the fraction susceptible s(t). The fraction susceptible is estimated from the cumulative age-specific hospitalizations at time t divided by age-specific hospitalization rates, which were in turn estimated from seroprevalences after the first wave in The Netherlands [3]. The explanatory variables for seasonal changes are absolute humidity (X_{AH})and temperature in degrees Celsius (X_{temp}) [4]. Additional explanatory variables that may affect R_t were also included: percent change in mobility from "workplace" and "retail and recreation" ($X_{mobility}$)[5], increased transmissibility due to change in circulating virus variants (notably alpha and delta) ($X_{variant}$)[6], day of the week (X_{day}), and intervention period ($X_{intervention}$). Intervention period is a variable indicating 2-week to 2-month time periods during the epidemic without change in control measures. New periods started 2020-03-13, 2020-05-06, 2020-06-01, 2020-07-01, 2020-09-01, 2020-09-29, 2020-10-26, 2020-12-21, 2021-01-23, 2021-02-08, 2021-03-01, 2021-04-26, 2021-05-17, 2021-06-05. The seasonality curve was extracted from the intercept and seasonal variable coefficients only, from which the amplitude was determined by fitting a sinusoidal curve.

Tables and Figures

Table S1. Vaccine effectiveness (VE) for each vaccine by dose based on observational studies.

Outcome	Vaccine[a]	Age Group	VE dose 1	VE dose 2	Reference
**Infection**	Pfizer/BioNTech	0-11	57%	88%	[7,8]
		12-29	57%	88%	
		30-49	57%	84%	
		50-69	57%	83%	
		70+	57%	73%	
	Moderna	0-11	66%	90%	
		12-29	66%	90%	
		30-49	66%	87%	
		50-69	66%	89%	
		70+	66%	86%	
	AstraZeneca	0-11	41%	73%	
		12-29	41%	73%	
		30-49	41%	76%	
		50-69	41%	79%	
		70+	41%	76%	
	Janssen	0-11	66%	--	
		12-29	66%	--	
		30-49	70%	--	
		50-69	73%	--	
		70+	78%	--	
**Hospitalization[b]**	Pfizer/BioNTech	0-11	95%	98%	[9]
		12-29	95%	98%	
		30-49	95%	98%	
		50-69	94%	97%	
		70+	85%	88%	
	Moderna	0-11	92%	97%	
		12-29	92%	97%	
		30-49	92%	97%	
		50-69	91%	96%	
		70+	86%	91%	
	AstraZeneca	0-11	85%	90%	
		12-29	85%	90%	
		30-49	85%	90%	
		50-69	87%	93%	
		70+	69%	74%	
	Janssen	0-11	94%	--	
		12-29	94%	--	
		30-49	94%	--	
		50-69	95%	--	
		70+	95%	--	
**Transmission**	All	All	46%	40%	[10]

[a] A delay of protection is assumed to be 14 days after the first dose and 7 days after the second dose for the Pfizer/BioNTech, Moderna, and AstraZeneca vaccines and 28 days after the first dose of the Janssen vaccine.

[b] VE against hospitalization is incorporated as a multiplier on the probability of being hospitalized after infection as $(1 - VE_{hospitalization})/(1-VE_{infection})$ to account for the inclusion of people who are never infected (and thus never hospitalized) included in the estimation of VE against hospitalization.

Table S2. Timeline of measures and advice during the COVID-19 outbreak in the Netherlands.

Date	Control Measures
16/3/2020	1.5 meters distance rule begins<br>All restaurants and bars are closed<br>Sport/fitness clubs, saunas, sex clubs and coffeeshops are closed<br>Schools and daycare facilities are closed.
23/3/2020-24/3/2020	Stay at home strongly encouraged<br>Maximum of 3 visitors at home and keep distance<br>Contact professions close<br>All gatherings will be banned, except funerals, weddings and religious gatherings (maximum of 30 persons, 1.5 meter distance)
11/5/2020	Schools in primary education and day care can re-open with reduced capacity<br>Contact professions and libraries can re-open<br>Masks advised on public transport
1/6/2020	Masks mandatory on public transport<br>Terraces re-open with 1.5 meter distance<br>Cinemas, restaurants and cafes, and cultural re- open with maximum 30 persons (incl. staff) and 1.5 meters distance<br>Secondary school partly re-open<br>Vaccination begins
8/6/2020	Primary education reopens 100%.
1/7/2020	Secondary schools re-open at 100% capacity<br>Fitness clubs, saunas, wellness centers, and casinos re-open<br>The maximum number of visitors expanded to 100 people for cinemas, restaurants and cafes, cultural institutions
6/8/2020	Colleges and universities can open for small groups
18/8/2020	Allowed to receive a maximum of 6 people in the excluding children < 13 years old<br>Home quarantine reduced from 14 to 10 days
20/9/2020	Large gathers limited to 50 people<br>Some hospitality sectors must close at midnight or 1 AM
29/9/2020	Maximum 3 visitors allowed at home<br>Large groups limited to 30 people<br>Face masks advised for people >12 years old in public indoor areas
14/10/2020	Can receive a maximum of 3 visitors at home per day<br>All cafes and restaurants close<br>Retail stores close at 8 PM<br>No team sports
23/10/2020	Hotels are not allowed to sell alcohol after 20.00
4/11/2020	Can receive maximum of 2 visitors at home per day.<br>Large groups, such as weddings, limited to 20 people
19/11/2020	Can receive a maximum of 3 people or 1 group at home per day.<br>Large gathers limited to 30 people<br>Work from home, unless there is no other option.
1/12/2020	Face masks mandatory on all public and indoor areas, in education, public transport and in contact professions.
15/12/2020	Only go outside with your household or in a group of max 2 people<br>Receive a maximum of 2 people at home aged 13 or older<br>Non-essential shops close
20/1/2021 – 23/1/2021	A curfew is introduced. Everyone stays indoors from 9 pm to 4.30 am.<br>Do not travel or book trips that take place up to and including March 31.
8/2/2021	Primary schools, childcare and special education reopen.<br>Out-of-school care (BSO) will remain closed
1/3/2021 – 3/3/2021	Secondary education and MBO will partly re-open<br>Contact professions re-open<br>People <27 years old can exercise together outside
16/3/2021	Retail stores re-open with reduced capacity
31-3-2021	The curfew will start at 10 PM instead of 9 PM.<br>Stores that mainly sell food, such as supermarkets, close at 9:45 PM instead of 8:45 PM.
19-4-2021	The out-of-school care will be fully open again
28-4-2021	The curfew is canceled

Date	Control Measures
	Can receive a maximum of 2 people at home per day.<br>Outdoor terraces open from 12:00 to 18:00 with maximum occupancy of 50 guests at 1.5 meter distance<br>Retail stores re-open for walk-in shopping (no reservation needed)
19-5-2021	From the age of 27 sports outside in groups allowed at a distance of 1.5 meters. Max. 30 people per group.<br>Indoor sports venues re-open<br>Non-essential travel allowed within the Netherlands.<br>Outdoor performance venues re-open<br>Terraces can be open from 6:00 to 20:00<br>Sex workers allowed to resume their profession<br>Libraries re-open
5-6-2014	4 visitors allowed at home per day.<br>Museums and cultural institutions re-open<br>Restaurants re-open<br>Large groups limited to 50 people
22-6-2021	Vaccination of 12-17 year olds begin

This is an abbreviated version of the timeline published at: https://www.rijksoverheid.nl/onderwerpen/coronavirus-tijdlijn

Table S3. Model parameters that do not vary with age.

Parameter	Description	Value	Details
β	Transmission rate	Varies over time	
σ	Inverse of the latent period	0.5/day	This results in a latent period of 2 days
γ	Inverse of the infectious period	0.5/day	This results in an infectious period of 2 days
λ	Force of infection	Varies over time	
$\boldsymbol{\epsilon}$	Rate of case importation	0.0/day	
α	Rate of vaccination with the first dose	This depends on the vaccine allocation schedule and varies over time	Calculated as a composite rate of multiple vaccines
$\boldsymbol{\alpha_2}$	Rate of vaccination with the second dose	This depends on the vaccine allocation schedule and varies over time	Calculated as a composite rate of multiple vaccines
$\boldsymbol{\delta_1}$	Delay to protection of first dose	See Table S1	With multiple vaccines, the weighted average is used
$\boldsymbol{\delta_2}$	Delay to protection of second dose	See Table S1	With multiple vaccines, the weighted average is used
η	1 – vaccine efficacy of first dose	See Table S1	With multiple vaccines, the weighted average is used
$\boldsymbol{\eta_2}$	1 – vaccine efficacy of second dose	See Table S1	With multiple vaccines, the weighted average is used

Table S4. Age-dependent model parameters.

Parameter	Description	Age group	Value	Details
h	Rate from infectious to hospital	0-9	0.0015	Calculated as the probability of infection to hospital divided by time from symptoms to hospital: $0.00347/2.29$
		10-19	0.0001	$0.000377/5.51$
		20-29	0.0002	$0.000949/5.05$
		30-39	0.0007	$0.00388/5.66$
		40-49	0.0013	$0.00842/6.55$
		50-59	0.0028	$0.0165/5.88$
		60-69	0.0044	$0.0251/5.69$
		70-79	0.0097	$0.0494/5.09$
		80+	0.0107	$0.0463/4.33$
i_1	Rate from hospital ward to IC	0-9	0.0000	Calculated as the probability of IC admission from hospital divided by average time from hospital admission to IC admission (2.28 days)
		10-19	0.0271	
		20-29	0.0422	
		30-39	0.0482	
		40-49	0.0719	
		50-59	0.0886	
		60-69	0.1070	
		70-79	0.0860	
		80+	0.0154	
i_2	Rate from IC back to hospital ward	0-9	0.0555	Calculated as the probability of admission back to hospital ward from IC divided by average length of stay in IC (15.6 days)
		10-19	0.0555	
		20-29	0.0555	
		30-39	0.0555	
		40-49	0.0555	
		50-59	0.0531	
		60-69	0.0080	
		70-79	0.0367	
		80+	0.0356	
d	Rate from hospital (before IC) to death	0-9	0.0003	Calculated as the probability of death from hospital admission divided by average length of time in hospital before death (7 days)
		10-19	0.0006	
		20-29	0.0014	
		30-39	0.0031	
		40-49	0.0036	
		50-59	0.0057	
		60-69	0.0151	
		70-79	0.0327	
		80+	0.0444	
d_{ic}	Rate from IC to death	0-9	0.0071	Calculated as the probability of death from IC divided by average length of time in IC before death (19 days)
		10-19	0.0071	
		20-29	0.0071	
		30-39	0.0071	
		40-49	0.0071	
		50-59	0.0090	
		60-69	0.0463	
		70-79	0.0225	
		80+	0.0234	
d_{hic}	Rate from hospital (after IC) to death	0-9	0.0000	Calculated as the probability of death from hospital ward (after IC) divided by average length of time in hospital ward (after IC) before death (10 days)
		10-19	0.0000	
		20-29	0.0000	
		30-39	0.0000	
		40-49	0.0000	
		50-59	0.0010	
		60-69	0.0040	
		70-79	0.0120	

Parameter	Description	Age group	Value	Details
		80+	0.0290	
r	Rate of recovery from hospital (before IC)	0-9	0.1263	Calculated as 1 – the probability of death from hospital admissions divided by the average time from hospital admission to discharge (7.9 days)
		10-19	0.1260	
		20-29	0.1254	
		30-39	0.1238	
		40-49	0.1234	
		50-59	0.1215	
		60-69	0.1132	
		70-79	0.0976	
		80+	0.0872	
r_{ic}	Rate of recovery from hospital (after IC)	0-9	0.0857	Calculated as 1 – the probability of death from hospital ward after IC divided by the average time from hospital ward (after IC) to discharge (10.1 days)
		10-19	0.0857	
		20-29	0.0857	
		30-39	0.0857	
		40-49	0.0857	
		50-59	0.0821	
		60-69	0.0119	
		70-79	0.0567	
		80+	0.0550	
$p_{ascertainment}$	Case ascertainment proportion	0-9	0.2900	
		10-19	0.3630	
		20-29	0.3810	
		30-39	0.5450	
		40-49	0.6450	
		50-59	0.5640	
		60-69	0.3650	
		70-79	0.3300	
		80+	0.4090	
**Relative Susceptibility/ Infectiousness**		0-9	1.000	
		10-19	3.051	
		20-29	5.751	
		30-39	3.538	
		40-49	3.705	
		50-59	4.365	
		60-69	5.688	
		70-79	5.324	
		80+	7.211	

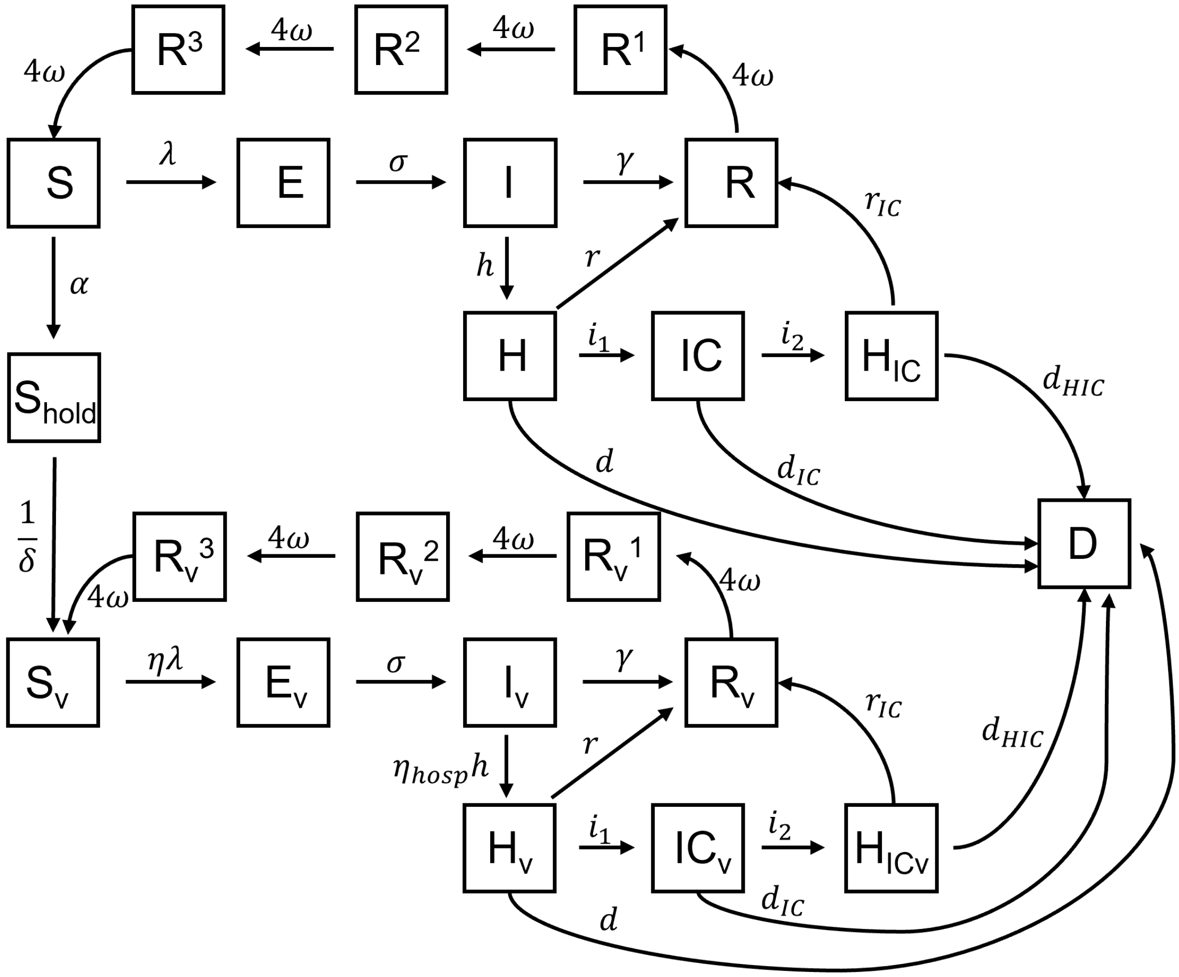

Figure S1. Basic conceptual model diagram. This diagram does not include the additional states after the first set vaccinated states or the age structure in the model. S = susceptible, E = exposed, I = infectious, R = recovered, R^x = additional recovered state x, H = hospitalized, IC = in intensive care, HIC = return to the hospital ward following treatment in IC, S_{hold} = vaccinated, but not yet protected, D = dead. States with subscript V indicate individuals who are vaccinated and protected by vaccination. λ = force of infection, σ = rate from exposed to infections, γ= rate from infectious to recovered, ω = rate of waning from recovered back to susceptible, α=rate of vaccination, δ=delay period to protection from vaccination, η=protection from vaccination against infection, η_{hosp}=protection from vaccination against hospitalization, h=rate from infectious to hospital, i_1=rate from hospital to intensive care, i_2=rate from intensive care back to hospital ward, d=rate from hospital to death, d_{IC}=rate from intensive care to death, d_{HIC}=rate from hospital ward after intensive care to death, r= rate of recovery from hospital, r_{IC}= rate of recovery from hospital ward after intensive care.

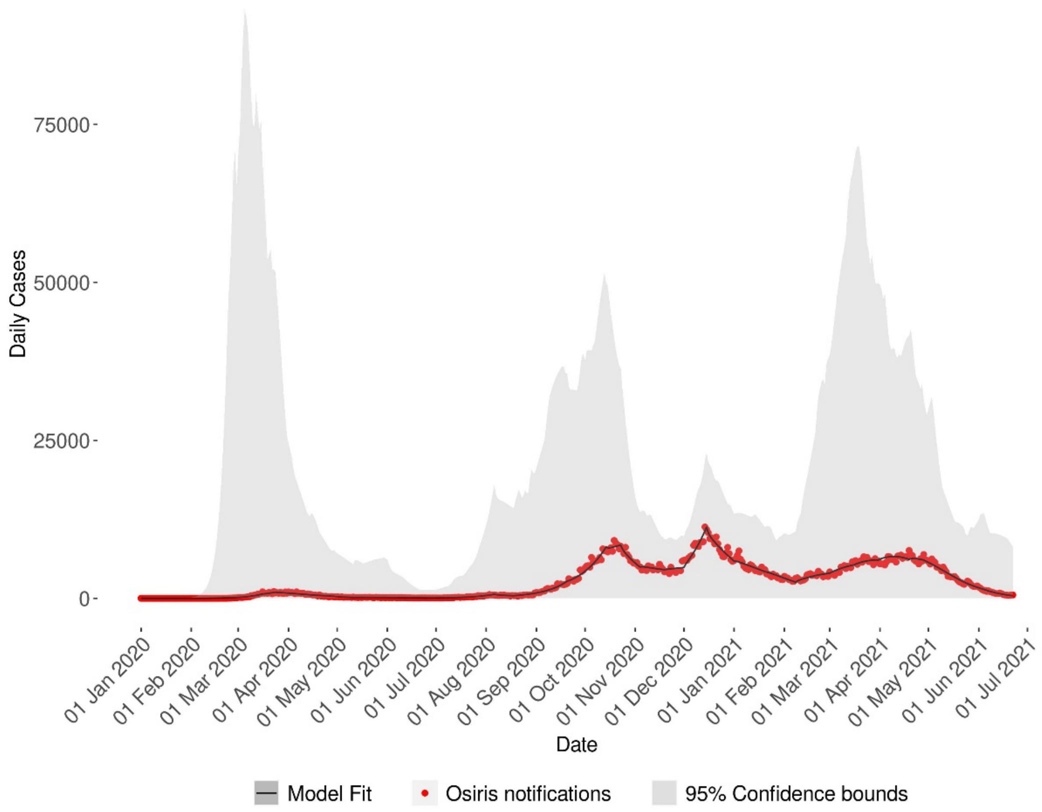

Figure S2. Fit to case notification data with 95% confidence bounds

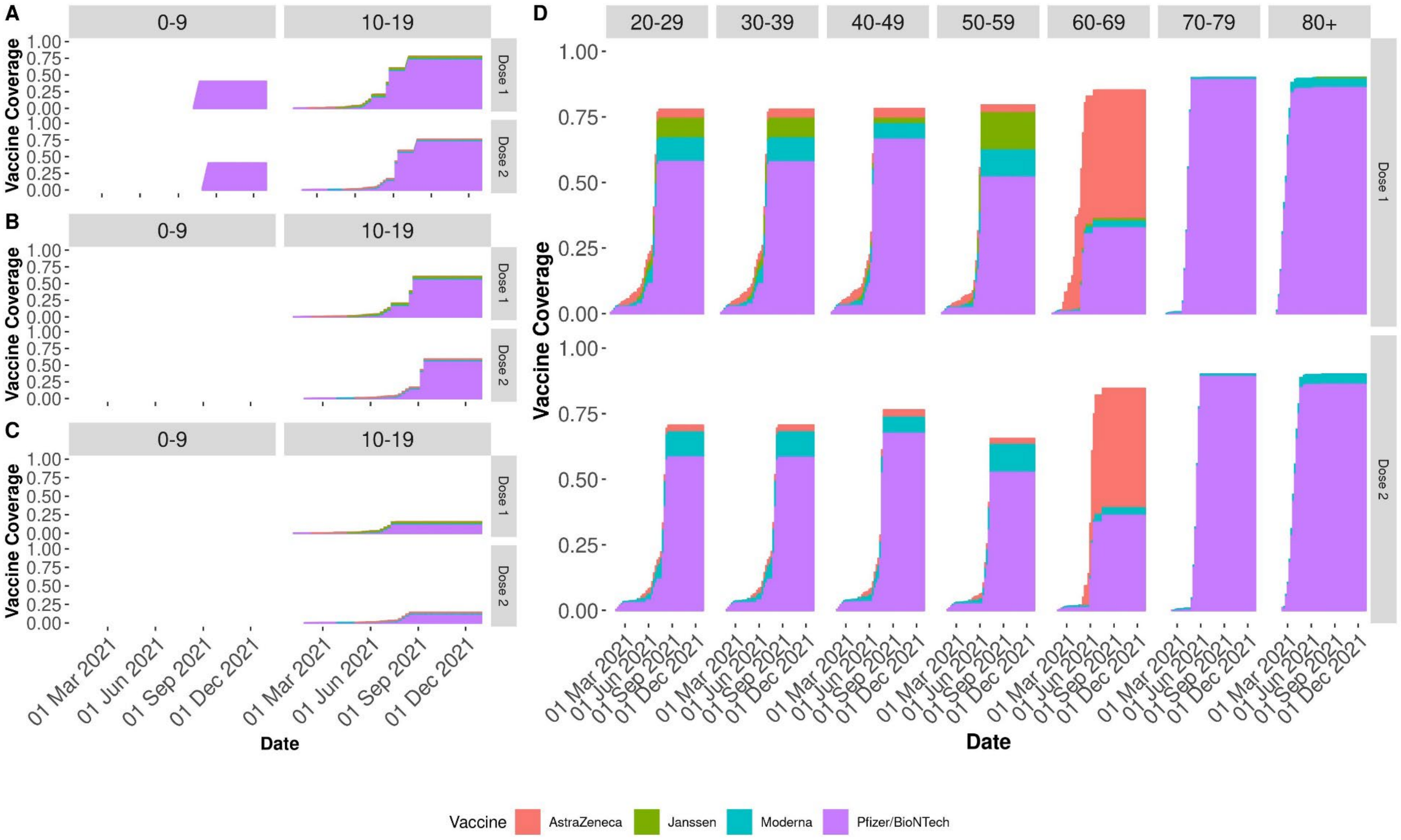

Figure S3. Vaccination coverage over time by dose, vaccine type, and age group for the different vaccination scenarios. A) vaccination coverage in 0-9 and 10-19 year old when everyone 5 years and above (5+) is vaccinated. B) vaccination coverage in 0-9 and 10-19 year old when everyone 12 years and above (12+) is vaccinated. C) vaccination coverage in 0-9 and 10-19 year old when everyone 18 years and above (18+) is vaccinated. D) vaccination coverage for adult age groups, (vaccination coverage in adults 20 years and above do not vary by vaccination scenario).

References

1. Rijksinstituut voor Volksgezondheid en Milieu (RIVM). Figures on the COVID-19 vaccination programme. Bilthoven: RIVM; 5 Oct 2021 [cited 8 Oct 2021]. Available: https://www.rivm.nl/en/covid-19-vaccination/figures-vaccination-programme

2. Liu X, Huang J, Li C, Zhao Y, Wang D, Huang Z, et al. The role of seasonality in the spread of COVID-19 pandemic. Environ Res. 2021;195: 110874.

3. Rijksinstituut voor Volksgezondheid en Milieu (RIVM). How calculation models contribute to the fight against COVID-19 [Dutch]. RIVM; 1 Oct 2021 [cited 18 Oct 2021]. Available: https://www.rivm.nl/coronavirus-covid-19/rekenmodellen#rm-hoe-bereken-je-de-r-454461-more

4. The Royal Netherlands Meteorological Institute (KNMI). Daily weather data in the Netherlands. De Bilt, The Netherlands: KNMI; 2021 [cited 18 Oct 2021]. Available: https://www.knmi.nl/nederland-nu/klimatologie/daggegevens

5. Google LLC. Google COVID-19 Community Mobility Reports. 2021. Available: https://www.google.com/covid19/mobility/

6. Rijksinstituut voor Volksgezondheid en Milieu (RIVM). Pathogen Surveillance. Bilthoven, The Netherlands: RIVM; 28 Sep 2021 [cited 18 Oct 2021]. Available: https://www.rivm.nl/en/coronavirus-covid-19/research/pathogen-surveillance

7. Martínez-Baz I, Trobajo-Sanmartín C, Miqueleiz A, Guevara M, Fernández-Huerta M, Burgui C, et al. Product-specific COVID-19 vaccine effectiveness against secondary infection in close contacts, Navarre, Spain, April to August 2021. Eurosurveillance. 2021;26: 2100894.

8. RIVM COVID-19 epidemiologie en surveillance team. Effectiviteit van COVID-19-vaccinatie tegen SARS-CoV-2 infectie in de Delta periode. Bilthoven: RIVM; 2021 Dec. Available: https://www.rivm.nl/sites/default/files/2021-12/Vaccineffectiviteit-teststraten%2020211216.pdf

9. RIVM COVID-19 epidemiologie en surveillance team. Effectiviteit van COVID-19 vaccinatie tegen ziekenhuis en intensive-care-opname in Nederland (opnames 11 juli – 23 november 2021). Bilthoven: RIVM; 2021 Nov. Available: https://www.rivm.nl/sites/default/files/2021-11/Analyse%20VE_Update_def%20-%20versie%2029%20nov%202021.pdf

10. de Gier B, Andeweg S, Backer JA, RIVM COVID-19 surveillance and epidemiology team, Hahné SJ, van den Hof S, et al. Vaccine effectiveness against SARS-CoV-2 transmission to household contacts during dominance of Delta variant (B.1.617.2), the Netherlands, August to September 2021. Euro Surveill. 2021;26. doi:10.2807/1560-7917.ES.2021.26.44.2100977
